# Stress Hyperglycemia Does Not Affect Clinical Outcome of Diabetic Patients Receiving Intravenous Thrombolysis for Acute Ischemic Stroke

**DOI:** 10.3389/fneur.2022.903987

**Published:** 2022-06-13

**Authors:** Giovanni Merlino, Sara Pez, Yan Tereshko, Gian Luigi Gigli, Simone Lorenzut, Andrea Surcinelli, Mariarosaria Valente

**Affiliations:** ^1^Stroke Unit, Department of Neuroscience, Udine University Hospital, Udine, Italy; ^2^Clinical Neurology, Department of Neuroscience, Udine University Hospital, Udine, Italy; ^3^Dipartimento di Area Medica (DAME), University of Udine, Udine, Italy

**Keywords:** stress hyperglycemia, GAR index, premorbid diabetic status, acute ischemic stroke, outcome, intravenous thrombolysis

## Abstract

Although stress hyperglycemia represents a main risk factor for poor outcome among patients with acute ischemic stroke (AIS) undergoing recanalization therapy, we have limited information regarding a possible influence of the premorbid diabetic status on this association. We recruited consecutive patients admitted to the Udine University Hospital with AIS who were treated with intravenous thrombolysis (IVT) from January 2015 to September 2020. On the basis of the premorbid diabetic status, our sample was composed of 130 patients with and 371 patients without diabetes. The glucose-to-glycated hemoglobin ratio (GAR) was used to measure stress hyperglycemia. Patients were stratified into 3 groups by tertiles of GAR (Q1–Q3). The higher GAR index was, the more severe stress hyperglycemia was considered. Among diabetic patients we did not observe any significant association between severe stress hyperglycemia and outcome measures (three-month poor outcome: Q1, 53.7%; Q2, 53.5%; Q3, 58.7%; *p* = 0.854; three-month mortality: Q1, 14.6%; Q2, 9.3%; Q3, 23.9%; *p* = 0.165; symptomatic intracranial hemorrhage: Q1, 7.3%; Q2, 14%; Q3, 19.6%; *p* = 0.256). Differently, non-diabetic subjects with more severe stress hyperglycemia showed a higher prevalence of three-month poor outcome (Q1, 32.2%; Q2, 27.7%; Q3, 60.3%; *p* = 0.001), three-month mortality (Q1, 9.1%; Q2, 8.4%; Q3, 18.3%; *p* = 0.026), and symptomatic intracranial hemorrhage (Q1, 0.8%; Q2, 0.8%; Q3, 9.9; *p* = 0.001). After controlling for several confounders, severe stress hyperglycemia remained a significant predictor of three-month poor outcome (OR 2.1, 95% CI 1.03–4.28, *p* = 0.041), three-month mortality (OR 2.39, 95% CI 1.09–5.26, *p* = 0.029) and symptomatic intracranial hemorrhage (OR 12.62, 95% CI 1.5–106, *p* = 0.02) among non-diabetics. In conclusion, premorbid diabetic status seems to influence outcome in AIS patients receiving IVT. Indeed, odds of functional dependency, mortality and hemorrhagic complications were significantly increased in patients with more severe stress hyperglycemia only when they were not affected by diabetes.

## Introduction

Acute illness is able to activate the stress response mediated by the hypothalamic-pituitary-adrenal axis and the sympathoadrenal system. As a consequence, excessive gluconeogenesis, glycogenolysis and insulin resistance occur causing a transient increase in blood glucose levels known as stress hyperglycemia (1).

Although stress hyperglycemia represents a physiological response, several studies performed both in intensive care unit (ICU) and in hospitalized non-ICU patients showed a strong relationship between stress hyperglycemia and poor clinical outcome (2–5). Interestingly, the detrimental effects of stress hyperglycemia would seem to be related to the premorbid diabetic status of patients. Numerous studies demonstrated that the association between increasing mean or median blood glucose and mortality was much stronger among non-diabetic critically ill individuals than among diabetic ones (6–12). Thus, Krinsley et al. concluded a recent review suggesting that intensive insulin treatment in critically ill patients should be tailored by premorbid diabetic status (13).

Although stress hyperglycemia represents a main risk factor for poor outcome among patients with acute ischemic stroke (AIS) undergoing recanalization therapy (14, 15), we have limited information regarding a possible influence of the premorbid diabetic status on this association. Whereas Chen et al. demonstrated that stress hyperglycemia was associated with unfavorable outcome only in non-diabetic patients when treated with mechanical thrombectomy (MT) (16), similar data in AIS subjects receiving intravenous thrombolysis (IVT) are lacking. Thus, the aim of this study was to evaluate if the role of stress hyperglycemia as predictor of poor outcome is influenced by the premorbid diabetic status among AIS patients treated with IVT.

## Materials and Methods

### Study Participants

This study is a retrospective analysis that includes consecutive patients admitted to the Udine University Hospital with AIS who were treated with IVT from January 2015 to September 2020. In accordance with the indications and contraindications of international guidelines, we treated patients showing symptoms onset within 4.5 h with alteplase at dosage of 0.9 mg/kg over 1 h (17, 18). Based on the recent results of the EXTEND trial, nine patients with wake-up stroke and salvageable brain tissue at the computed tomography perfusion were treated beyond 4.5 h (19). AIS patients undergoing endovascular thrombectomy in addition to IVT were excluded. All patients or his/her representatives gave informed, signed consent to use of their data for research purposes. The study was approved by the local ethics committee (Ref. No. CEUR-2020-Os-173).

During the study period, 537 patients were treated with IVT for AIS. Of them, 36 with missing data on fasting glucose or HbA1c were excluded. The remaining 501 AIS patients were included in the study and distinguished in subjects with (*n* = 130, 25.9%) and without (*n* = 371, 74.1%) diabetes, on the basis of their premorbid diabetic status. In particular, we defined diabetic patients who reported a history of diabetes mellitus confirmed in medical records and/or who used insulin/oral hypoglycemic agents and/or who had HbA1c levels of 6.5% or more.

### Data Collection

We collected information on the following variables: age, sex, vascular risk factors, laboratory findings, systolic blood pressure at admission, and pharmacological treatment. In addition, early ischemic changes on native CT-scan within the middle cerebral artery were graded according to the ASPECT Score (20). Vascular risk factors were defined as follows: (1) previous transient ischemic attack/stroke was defined if the patient had a history of ischemic (transient attack or stroke) or hemorrhagic cerebrovascular disease; (2) the presence of cardiovascular disease was based on the history of previous ischemic heart disease and/or revascularization treatment using percutaneous coronary intervention/coronary artery bypass grafting; (3) atrial fibrillation was defined if the patient had past medical history of atrial fibrillation that had been confirmed in medical records; (4) high blood pressure was defined as the history of hypertension and/or use of antihypertensive medication; (5) a presence of hypercholesterolemia was based on the use of lipid-lowering medications; (6) information on active tobacco use was used for defining patient as a current smoker.

### Clinical Assessment

Stroke etiology was defined using the Trial of ORG 10,172 in Acute Stroke Treatment (TOAST) classification (21). The National Institute of Health Stroke Scale (NIHSS) score was adopted for determining stroke severity at admission and at discharge. We defined patients with major neurological improvement those who had an improvement of ≥ 8 points on the NIHSS from baseline or a NIHSS score of 0 or 1 at discharge. Functional outcome was assessed by means of the modified Rankin Scale (mRS) at admission, based on pre-stroke disability, and 3 months after stroke. The mRS score after discharge was recorded at the patients' routine clinical visit or through telephone interview with patients or their immediate caregivers. The mRS score was dichotomized into: favorable outcome (0–2) and poor outcome (3–6). The presence of intracranial hemorrhage (ICH) was defined as any parenchymal hematoma (PH) based on the European Cooperative Acute Stroke Study (ECASS) morphologic definitions (ECASS PH-1 or PH-2) (22), whereas presence of SICH was based on the ECASS-III protocol (23). Finally, we collected information on time from symptom onset to IVT and from hospital arrival to alteplase (door-to-needle time).

### Assessment of Stress Hyperglycemia

For laboratory tests, including fasting plasma glucose and HbA1c, venous blood samples were drawn within 24 h after hospitalization, during the morning hours (range: 06.00–08:00) after an overnight fast (at least 12 h). Stress hyperglycemia was estimated by the GAR index that was calculated using the following formula: fasting plasma glucose (mg/dl) / HbA1c (%). The median value of the GAR index among diabetic and non-diabetic patients was 19.4 (IQR 16–23.2) and 16.7 (IQR 14.9–18.9), *p* < 0.001, respectively. In order to investigate the clinical consequences of severe stress hyperglycemia, diabetic and non-diabetic patients were stratified into 3 groups by tertiles of GAR (Q1–Q3) for further comparisons. The first tertile included patients with GAR values lower than 33.3th percentile, i.e. lower than 17.3 for diabetics and 15.5 for non-diabetics; the second tertile included patients with GAR values within 33.3th to 66.6th percentile, i.e. comprised between 17.3–21.5 for diabetics and 15.5–17.8 for non-diabetics; the third tertile included patients with GAR values higher than 66.6th percentile, i.e. higher than 21.5 for diabetics and 17.8 for non-diabetics. The higher GAR index was, the more severe stress hyperglycemia was considered.

### Outcome Measures

The following primary endpoints were analyzed: three-month poor outcome, three-month all-cause mortality and presence of SICH. No major neurological improvement at discharge, in-hospital all-cause mortality and presence of ICH were used as secondary outcome measures. All the outcome measures were collected as part of our routine clinical practice in patients affected by cerebrovascular events.

### Statistical Analysis

Data are displayed in tables as mean and standard deviation, if not otherwise specified. Chi square test or the Fisher's exact test, when appropriate, were used for categorial variables. One-way analysis of variance for normally distributed continuous variables, and the Kruskal-Wallis test for non-normally distributed continuous variables and for ordinal variables were used. Post-hoc analysis was performed by means of the Bonferroni test or the Dunn test, when appropriate. The Kolmogorov–Smirnov test with Lilliefors significant correction was used to assess normal distribution of data.

Multiple logistic regression analysis was performed to assess the impact of stress hyperglycemia, as represented by GAR tertiles, on primary and secondary outcome measures with the lowest GAR tertile as reference. The regression model was adjusted for all potential confounders with a probability value <0.1 in univariate analysis. The specific variables considered for adjustment in each analysis are indicated in the legend of **Table 3** and Supplementary Table 1.

All probability values are two-tailed. A *p* value < 0.05 was considered statistically significant. Statistical analysis was carried out using the SPSS Statistics, Version 22.0 (Chicago, IL, USA).

## Results

### Baseline Characteristics

**Figure 1:**
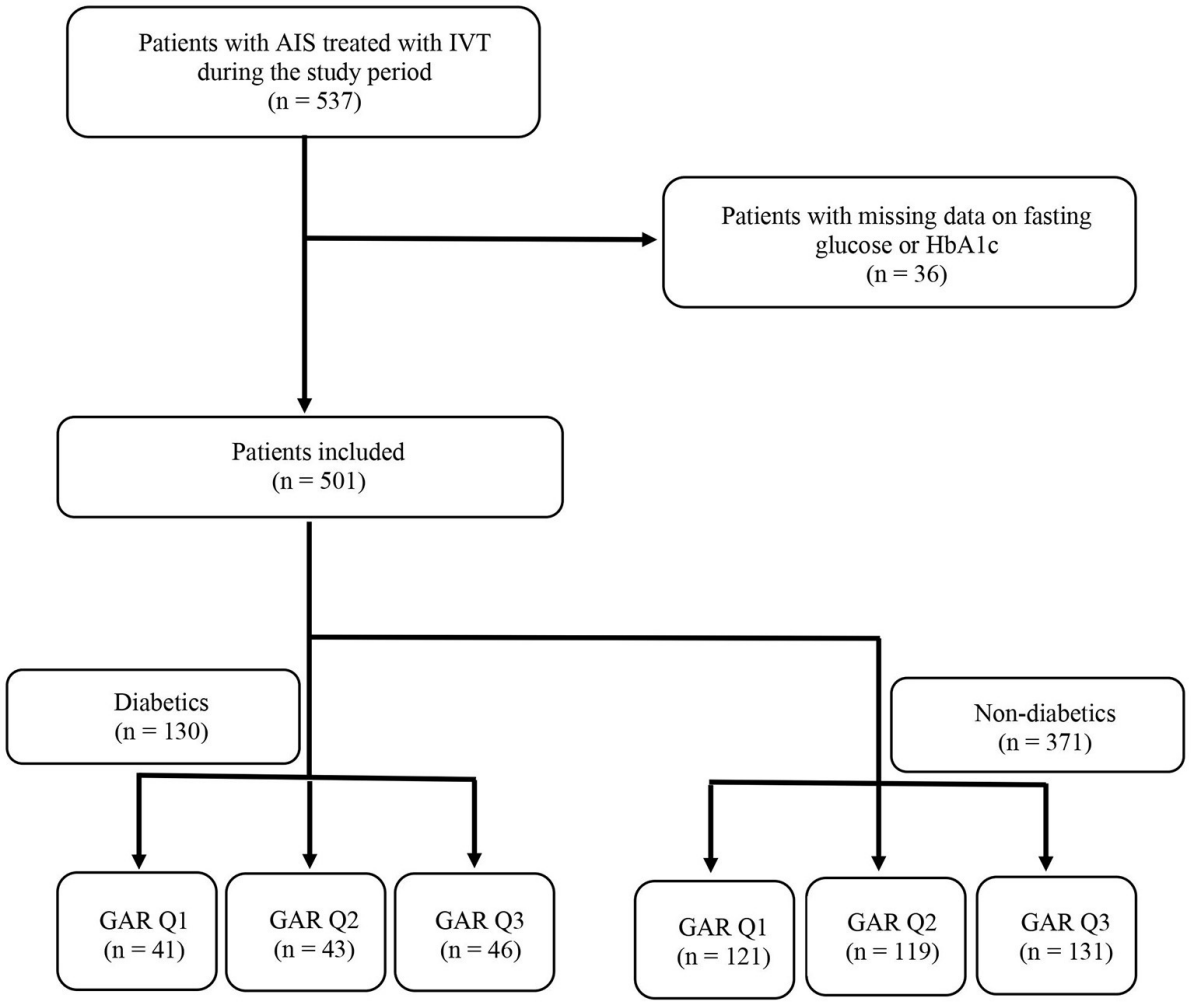
Flow diagram of the study. AIS, acute ischemic stroke; IVT, intravenous thrombolysis; HbA1c, glycated hemoglobin; GAR Q1, first glucose-to-glycated hemoglobin ratio tertile; GAR Q2, second glucose-to-glycated hemoglobin ratio tertile; GAR Q3, third glucose-to-glycated hemoglobin ratio tertile.

The patients, with and without diabetes, were divided into 3 tertiles according to GAR. As reported in the flow diagram of the study (see Figure 1), the number of diabetic patients included in each GAR tertile was the following: 41 patients (31.5%) in the first tertile, 43 patients (33.1%) in the second tertile and 46 patients (35.4%) in the third tertile. Whereas, non-diabetic patients were distributed as follows: 121 patients (32.6%) in the first tertile, 119 patients (32.1%) in the second tertile and 131 patients (35.3%) in the third tertile.

Table 1 reports baseline characteristics among the 130 patients with diabetes. We did not observe any significant difference regarding demographic features and vascular risk factors between the 3 tertiles of GAR. Regarding laboratory parameters, patients in the third GAR tertile had higher levels of C-reactive protein and fasting glucose than those in the other two tertiles. Finally, with respect to patients in the first GAR tertile, subjects in the third GAR tertile showed higher systolic blood pressure values and were treated less frequently with antiplatelets prior to hospital admission (see Table 1).

**Table 1:** General characteristics of 130 diabetic patients according to the GAR tertiles.

	**GAR Q1 (*n =* 41)**	**GAR Q2** **(*n =* 43)**	**GAR Q3 (*n =* 46)**	***p***
**Demographic data**				
Age, years^*^	76 (68–81.5)	74 (68–84)	76.5 (67.2–82.2)	0.992
Males, *n* (%)	29 (70.7)	25 (58.1)	25 (54.3)	0.269
**Vascular risk factors**				
Previous transient ischemic attack/stroke, *n* (%)	7 (17.1)	7 (16.3)	3 (6.5)	0.259
Cardiovascular disease, *n* (%)	12 (29.3)	6 (9.9)	12 (10.6)	0.208
Atrial fibrillation, *n* (%)	7 (17.1)	4 (9.3)	10 (21.7)	0.276
Hypertension, *n* (%)	35 (85.4)	36 (83.7)	34 (73.9)	0.334
Hypercholesterolemia, *n* (%)	17 (41.5)	14 (32.6)	16 (36.2)	0.677
Current smoking, *n* (%)	7 (17.1)	4 (9.3)	5 (10.9)	0.519
**Laboratory findings**				
Hb, g/dl	13.2 ± 1.4	13.1 ± 1.8	13 ± 1.8	0.902
Platelets, 10^3^/mmc^*^	196 (151–235.5)	178 (159.7–214.2)	198 (157.2–251.7)	0.353
aPTT ratio^*^	0.96 (0.87–1.08)	0.94 (0.86–1.04)	0.94 (0.88–1.1)	0.861
INR^*^	1.06 (1–1.16)	1.04 (0.99–1.13)	1.04 (0.99–1.11)	0.470
Creatinine, mg/dl^*^	0.93 (0.82–1.11)	0.84 (0.74–0.98)	0.9 (0.67–1.19)	0.246
C-reactive protein, mg/l^*^	2.48 (1.47–7.16)	2.76 (1.54–9.47)	7.66 (2.53–17.02)	0.011
Protein, g/dl^*^	6.2 (5.8–6.6)	6.4 (5.9–6.7)	6.3 (6.2–6.7)	0.874
Fasting plasma glucose, mg/dl^*^	102 (93–113.5)	131 (121–147)	178 (156.7–216)	0.001
HbA1c values, %^*^	6.9 (6.6–7.6)	6.9 (6.6–7.2)	7.1 (6.7–7.7)	0.369
GAR index^*^	15.1 (13.1–16)	19.2 (18–20.4)	24.5 (22.4–27.6)	0.001
Total cholesterol, mg/dl^*^	158 (133.5–175)	159 (121.2–186.5)	165 (149–179.5)	0.359
HDL cholesterol, mg/dl^*^	44 (34.5–54.5)	48.5 (40.7–59.2)	47 (41–60.5)	0.852
LDL cholesterol, mg/dl^*^	84 (66.5–103)	84.5 (61.5–112.2)	90 (74.5–117.5)	0.449
Triglycerides, mg/dl^*^	121 (90–178.7)	95.5 (70.5–124)	94 (73–140)	0.023
**Blood pressure**				
Systolic blood pressure, mmHg	150.7 ± 21.6	162.7 ± 26.1	169.2 ± 23.3	0.002
**Antithrombotic treatment at admission**				
Antiplatelets, *n* (%)	28 (68.3)	20 (46.5)	18 (39.1)	0.02
Anticoagulants, *n* (%)	1 (2.4)	1 (2.3)	1 (2.2)	0.997
**Antidiabetic drugs at admission**				
Oral hypoglycemics, *n* (%)	3 (7.3)	1 (2.4)	3 (6.7)	0.557
Insulin, *n* (%)	16 (39)	22 (52.4)	27 (60)	0.147
Median baseline ASPECTS (range)	10 (8–10)	10 (8–10)	10 (8–10)	1
**Stroke subtypes based on TOAST classification**				0.801
Large arterial atherosclerosis, *n* (%)	4 (9.8)	7 (16.3)	8 (17.4)	
Cardioembolism, *n* (%)	16 (39)	14 (32.6)	16 (34.8)	
Small vessel disease, *n* (%)	8 (19.5)	8 (18.6)	4 (8.7)	
Other determined etiology, *n* (%)	0 (0)	1 (2.3)	1 (2.2)	
Undetermined etiology, *n* (%)	13 (31.7)	13 (30.2)	17 (37)	
**Baseline clinical characteristics**				
Median NIHSS score at admission (IQR)	7 (4–15)	7 (4–18)	9 (4.75–18)	0.630
Median NIHSS score at discharge (IQR)	1 (0–4)	2.5 (0–10)	2 (0–8)	0.163
Median pre-stroke mRS (range)	0 (0–4)	0 (0–4)	0 (0–5)	0.414
**Information on IVT therapy**				
Time from symptoms onset to alteplase, min^*^	150 (130–210)	150 (120–183)	162 (118.7–206.2)	0.499
Door-to-needle time, min^*^	83 (58–110.5)	71 (50–92)	73.5 (55.7–89.5)	0.351

*GAR Q1, first glucose-to-glycated hemoglobin ratio tertile; GAR Q2, second glucose-to-glycated hemoglobin ratio tertile; GAR Q3, third glucose-to-glycated hemoglobin ratio tertile; Hb, hemoglobin; aPTT, activated partial thromboplastin time; INR, international normalized ratio; HbA1c, glycated hemoglobin; GAR, glucose-to-glycated hemoglobin ratio; HDL, high–density lipoprotein; LDL, low-density lipoprotein; ASPECTS, Alberta Stroke Programme Early CT Score; TOAST, Trial of ORG 10172 in Acute Stroke Treatment; NIHSS, National Institute of Health Stroke Scale; mRS, modified Rankin Scale; IVT, intravenous thrombolysis*.

Data are presented as mean and standard deviation for normally distributed continuous variables. Non-normally distributed continuous variables are displayed as median and interquartile range and are identified by an asterisk (^*^).

As reported in Table 2, non-diabetic patients in the third GAR tertile had higher levels of fasting glucose than those in the other two tertiles. Systolic blood pressure values at admission were different between non-diabetics in the first (lower values) and third (higher values) tertile of GAR. Despite door-to-needle time was shorter, non-diabetic patients in the third GAR tertile showed significantly higher NIHSS scores than subjects in the first GAR tertile both at admission and at discharge (see Table 2).

**Table 2:** General characteristics of 371 non–diabetic patients according to the GAR tertiles.

	**GAR Q1 (*n =* 121)**	**GAR Q2** **(*n =* 119)**	**GAR Q3 (*n =* 131)**	***P***
**Demographic data**				
Age, years^*^	76 (61–82)	72 (65–80)	76 (69–84)	0.194
Males, *n* (%)	54 (44.6)	64 (53.8)	73 (55.7)	0.176
**Vascular risk factors**				
Previous transient ischemic attack/stroke, *n* (%)	7 (17.1)	7 (16.3)	3 (6.5)	0.259
Cardiovascular disease, *n* (%)	15 (12.4)	8 (6.7)	11 (8.4)	0.292
Atrial fibrillation, *n* (%)	25 (20.7)	11 (9.2)	20 (15.3)	0.047
Hypertension, *n* (%)	70 (57.9)	69 (58)	87 (66.4)	0.277
Hypercholesterolemia, *n* (%)	34 (28.1)	30 (25.2)	27 (20.6)	0.377
Current smoking, *n* (%)	22 (18.2)	21 (17.6)	13 (9.9)	0.120
**Laboratory findings**				
Hb, g/dl	12.8 ± 2	13.5 ± 1.6	13.3 ± 1.7	0.002
Platelets, 10^3^/mmc^*^	206 (173.5–246.5)	196 (166–237)	194.5 (162–226)	0.142
INR^*^	1.04 (0.99–1.12)	1.05 (1–1.11)	1.06 (1–1.15)	0.406
Creatinine, mg/dl^*^	0.83 (0.72–0.97)	0.88 (0.75–1.04)	0.87 (0.72–1.02)	0.331
C–reactive protein, mg/l^*^	3.67 (1.63–8.69)	3.81 (1.63–9.44)	4.64 (2.04–11.62)	0.782
Protein, g/dl^*^	6.2 (5.8–6.5)	6.3 (6–6.8)	6.3 (6–6.8)	0.276
Fasting plasma glucose, mg/dl^*^	83 (78–86)	96 (92–101)	115 (104–134)	0.001
HbA1c values, %^*^	5.8 (5.6–6)	5.8 (5.6–6.1)	5.7 (5.4–6)	0.114
GAR index^*^	14.2 (13.6–14.9)	16.7 (16.1–17.1)	20.4 (18.8–22.5)	0.001
Total cholesterol, mg/dl	174 ± 40	182.1 ± 45.7	176.5 ± 42.2	0.343
HDL cholesterol, mg/dl^*^	52 (40.2–61.7)	54 (42–66)	55 (45–64.7)	0.488
LDL cholesterol, mg/dl	100.1 ± 36	105 ± 39.7	101.4 ± 37.3	0.597
Triglycerides, mg/dl^*^	93 (69.5–123)	96 (72–129)	83.5 (61.2–115.2)	0.065
**Blood pressure**				
Systolic blood pressure, mmHg^*^	155 (140–167)	159 (139.5–175)	165 (180–145.4)	0.013
**Antithrombotic treatment at admission**				
Antiplatelets, *n* (%)	36 (29.8)	29 (24.4)	40 (30.5)	0.508
Anticoagulants, *n* (%)	9 (7.4)	5 (4.2)	8 (6.1)	0.566
**Antidiabetic drugs at admission**				
Oral hypoglycemics, *n* (%)	0 (0)	0 (0)	0 (0)	1
Insulin, *n* (%)	0 (0)	0 (0)	0 (0)	1
Median baseline ASPECTS (range)	10 (9–10)	10 (8–10)	10 (7–10)	1
**Stroke subtypes based on TOAST classification**				0.172
Large arterial atherosclerosis, *n* (%)	13 (10.7)	13 (10.9)	22 (16.8)	
Cardioembolism, *n* (%)	44 (36.4)	39 (32.8)	55 (42)	
Small vessel disease, *n* (%)	13 (10.7)	11 (9.2)	14 (10.7)	
Other determined etiology, *n* (%)	3 (2.5)	5 (4.2)	7 (5.3)	
Undetermined etiology, *n* (%)	48 (39.7)	51 (42.9)	33 (25.2)	
**Baseline clinical characteristics**				
Median NIHSS score at admission (IQR)	6 (4–9.5)	6 (4–9)	8 (5–16)	0.046
Median NIHSS score at discharge (IQR)	1 (0–3)	1 (0–3)	4 (1–10)	0.001
Median pre-stroke mRS (range)	0 (0–5)	0 (0–4)	0 (0–5)	0.106
**Information on IVT therapy**				
Time from symptoms onset to alteplase, min^*^	151 (113.7–198.7)	157.5 (129–195)	156 (125–200)	0.835
Door-to-needle time, min^*^	75 (59.2–106.5)	77 (57.5–100.2)	67 (53.5–80)	0.006

*GAR Q1, first glucose-to-glycated hemoglobin ratio tertile; GAR Q2, second glucose-to-glycated hemoglobin ratio tertile; GAR Q3, third glucose-to-glycated hemoglobin ratio tertile; Hb, hemoglobin; aPTT, activated partial thromboplastin time; INR, international normalized ratio; HbA1c, glycated hemoglobin; GAR, glucose-to-glycated hemoglobin ratio; HDL, high–density lipoprotein; LDL, low–density lipoprotein; ASPECTS, Alberta Stroke Programme Early CT Score; TOAST, Trial of ORG 10172 in Acute Stroke Treatment; NIHSS, National Institute of Health Stroke Scale; mRS, modified Rankin Scale; IVT, intravenous thrombolysis*.

Data are presented as mean and standard deviation for normally distributed continuous variables. Non-normally distributed continuous variables are displayed as median and interquartile range and are identified by an asterisk (^*^).

### Association of Stress Hyperglycemia With Clinical Outcomes in Univariate Analysis

**Figure 2:**
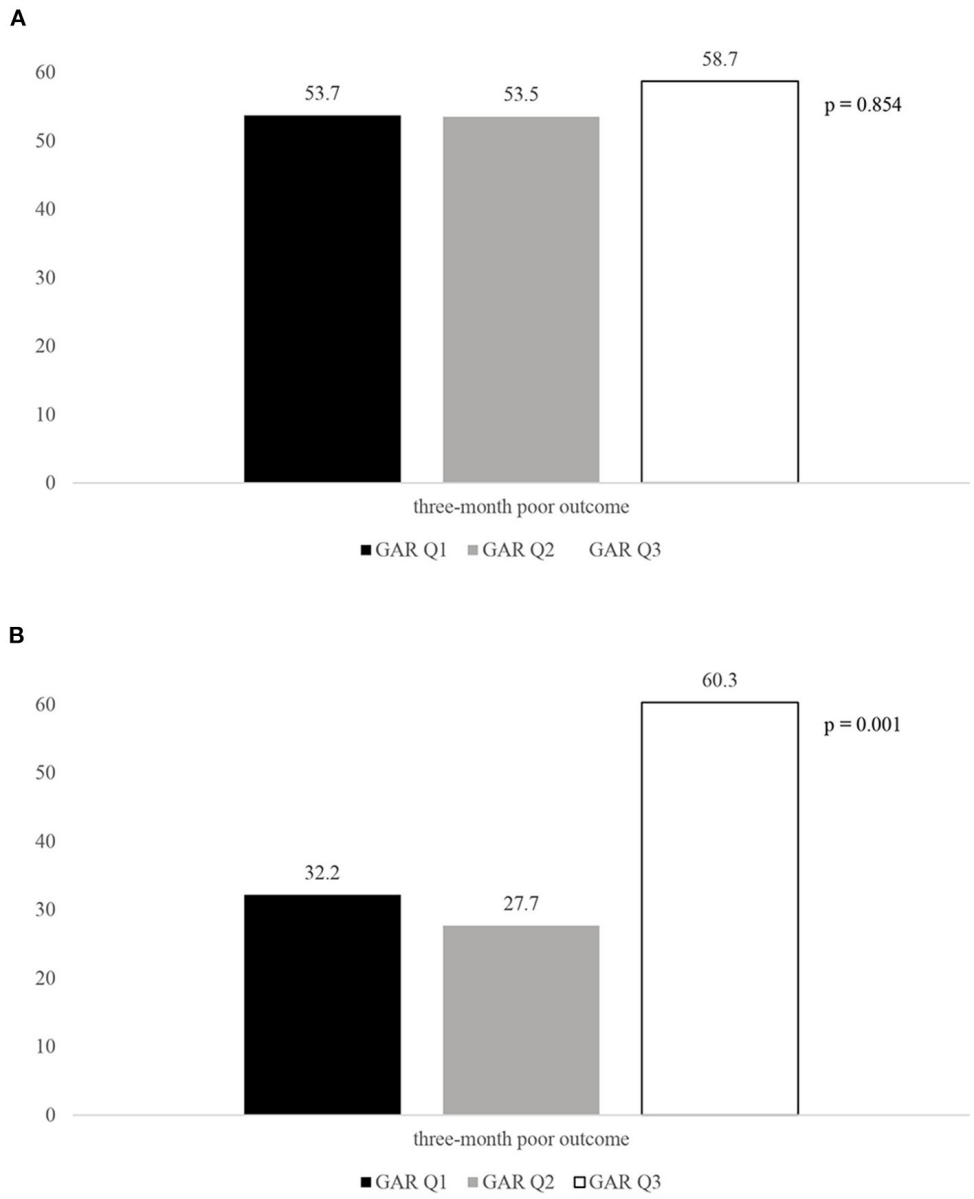
Rates of three-month poor outcome according to the GAR tertiles in diabetic. **(A)** and non-diabetic patients **(B)**. GAR Q1, first glucose-to-glycated hemoglobin ratio tertile; GAR Q2, second glucose-to-glycated hemoglobin ratio tertile; GAR Q3, third glucose-to-glycated hemoglobin ratio tertile.

**Figure 3:**
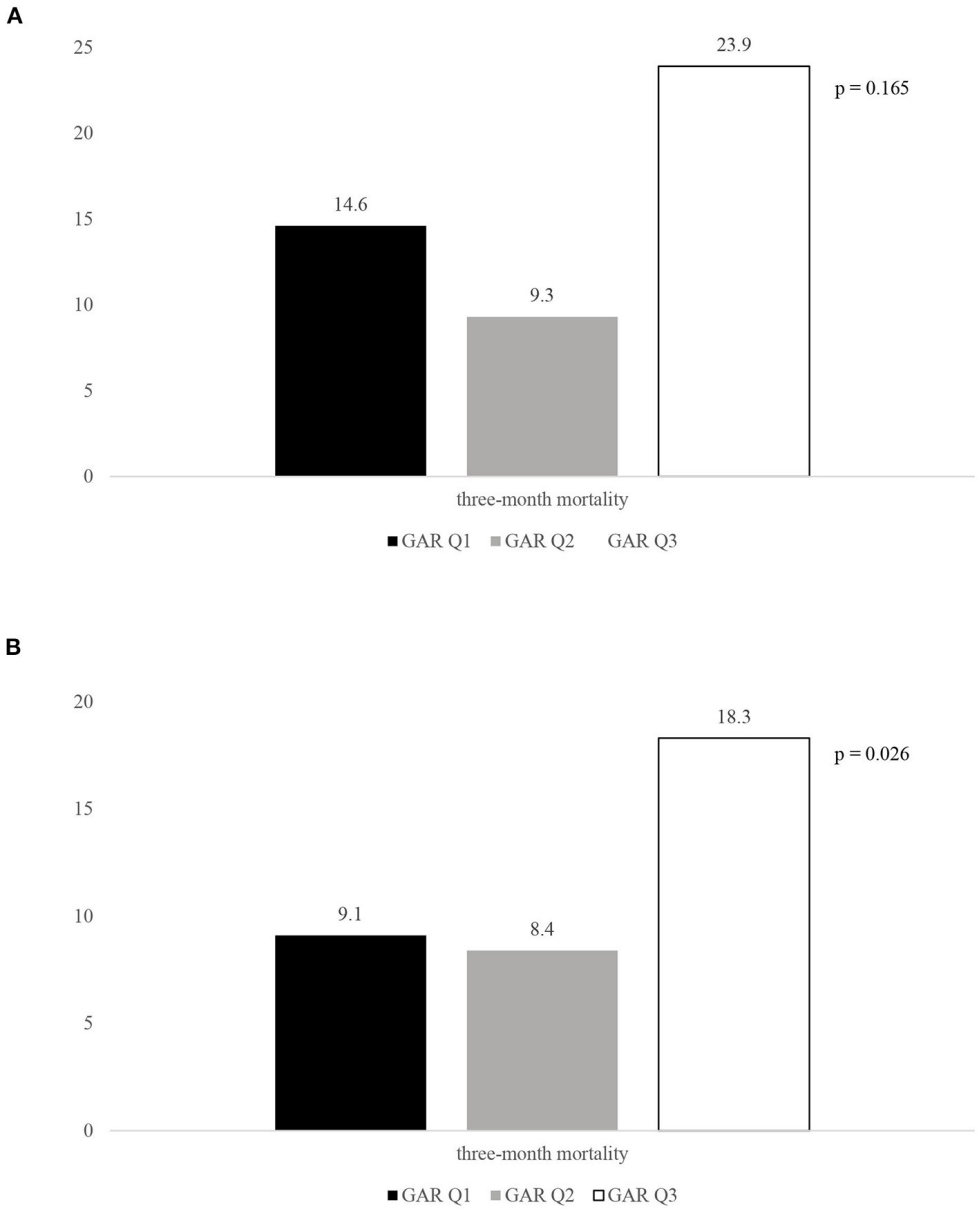
Rates of three-month mortality according to the GAR tertiles in diabetic. **(A)** and non-diabetic patients **(B)**. GAR Q1, first glucose-to-glycated hemoglobin ratio tertile; GAR Q2, second glucose-to-glycated hemoglobin ratio tertile; GAR Q3, third glucose-to-glycated hemoglobin ratio tertile.

**Figure 4:**
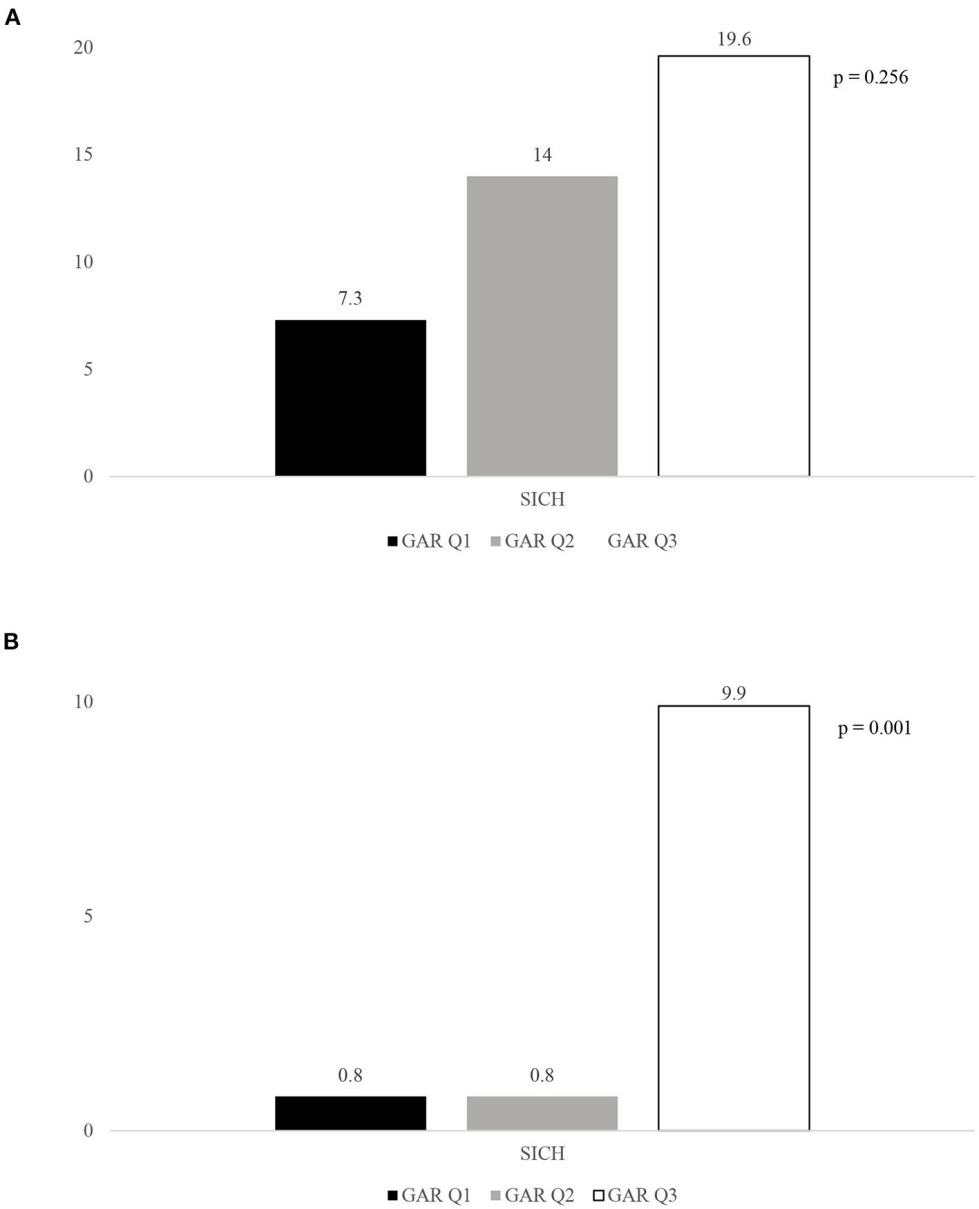
Rates of SICH according to the GAR tertiles in diabetic. **(A)** and non-diabetic patients **(B)**. GAR Q1, first glucose-to-glycated hemoglobin ratio tertile; GAR Q2, second glucose-to-glycated hemoglobin ratio tertile; GAR Q3, third glucose-to-glycated hemoglobin ratio tertile; SICH, symptomatic intracranial hemorrhage.

Rates of 3-month poor outcome, 3-month all-cause mortality and presence of SICH according to GAR tertiles in diabetic and non-diabetic patients are reported in Figures 2–4. Differently from diabetic patients, we observed a significant association between the higher tertile of GAR and primary endpoints in non-diabetic patients. Similar results were obtained for secondary outcome measures (see Supplementary Figures 1–3 in Data Supplement).

### Association of Stress Hyperglycemia With Clinical Outcomes in Multivariate Analysis

As reported in Table 3, for primary outcome measures, and in Supplementary Table 1, for secondary outcome measures, severe stress hyperglycemia was not associated with 3-month poor outcome (OR 0.82, 95% CI 0.29–2.28, *p* = 0.820), 3-month mortality (OR 3.17, 95% CI 0.66–15.26, *p* = 0.15), SICH (OR 2.72, 95% CI 0.72–10.23, *p* = 0.14), no major neurological improvement at discharge (OR 1.63, 95% CI 0.62–4.3, *p* = 0.322), in-hospital mortality (OR 18.29, 95% CI 0.53–63.38, *p* = 0.107) and ICH (OR 2.99, 95% CI 0.74–12.08, *p* = 0.123) among diabetic patients. Differently, the highest GAR tertile was an independent predictor of all the endpoints among non-diabetic subjects (3-month poor outcome: OR 2.83, 95% CI 1.37–635.87, *p* = 0.005; 3-month mortality: OR 2.47, 95% CI 1.08–5.66, *p* = 0.033; SICH: OR 10.79, 95% CI 1.28–90.63, *p* = 0.029; no major neurological improvement at discharge: OR 2.11, 95% CI 1.15–3.87, *p* = 0.016; in-hospital mortality: OR 5.19, 95% CI 1.05–25.56, *p* = 0.043; ICH: OR 5.19, 95% CI 1.05–25.61, *p* = 0.043).

**Table 3:** Logistic regression model: adjusted ORs (95% CIs) of the GAR tertiles in relation to the respective primary outcome measures in 130 diabetic patients and in 371 non–diabetic ones.

	**GAR Q1**	**GAR Q2**	**GAR Q3**
**Diabetics**, ***n =*** **130**			
Three-month poor outcome^†^	1	0.93 (0.33–2.63) *p =* 0.929	0.82 (0.29–2.28) *p =* 0.82
Three-month mortality^††^	1	0.64 (0.08–4.86) *p =* 0.669	3.17 (0.66–15.26) *p =* 0.15
Presence of SICH^†††^	1	0.28 (0.04–1.78) *p =* 0.179	2.72 (0.72–10.23) *p =* 0.14
**Non-diabetics**, ***n =*** **371**			
Three-month poor outcome^*^	1	0.99 (0.47–2.08) *p =* 0.978	2.83 (1.37–5.87) *p =* 0.005
Three-month mortality^**^	1	1.34 (0.52–3.47) *p =* 0.54	2.47 (1.08–5.66) *p =* 0.033
Presence of SICH^***^	1	1.43 (0.08–24.5) *p =* 0.805	10.79 (1.28–90.63) *p =* 0.029

*GAR Q1, first glucose-to-glycated hemoglobin ratio tertile; GAR Q2, second glucose-to-glycated hemoglobin ratio tertile; GAR Q3, third glucose-to-glycated hemoglobin ratio tertile; SICH, symptomatic intracranial hemorrhage*.

† *Adjusted for: age, stroke due to cardioembolism, stroke due to small vessel disease, baseline NIHSS score and pre-stroke mRS*.

†† *Adjusted for: age, atrial fibrillation, creatinine, C-reactive protein, total cholesterol, LDL cholesterol, stroke due to small vessel disease and baseline NIHSS score*.

††† *Adjusted for: protein, total cholesterol, stroke due to small vessel disease and baseline NIHSS score*.

* *Adjusted for: age, sex, atrial fibrillation, C-reactive protein, ASPECTS, stroke due to large arterial atherosclerosis, baseline NIHSS score and pre-stroke mRS*.

** *Adjusted for: age: sex, atrial fibrillation, creatinine, C-reactive protein, use of antiplatelets at admission, baseline NIHSS score and pre-stroke mRS*.

*** *Adjusted for, age, atrial fibrillation, creatinine, triglycerides, baseline NIHSS score and pre-stroke mRS*.

## Discussion

For the first time, we demonstrated that premorbid diabetic status influences the detrimental effects of stress hyperglycemia in AIS patients treated with alteplase. In fact, clinical outcome was significantly worsened by high levels of stress hyperglycemia only in patients not affected by diabetes.

In 2008 Egi et al. performed a retrospective observational study including 4,946 critically ill patients. The authors compared the relationship between acute glycemia during ICU stay and mortality in diabetic and non-diabetic patients. Subjects without diabetes with a time-weighted glucose concentration between 8 and 10.0 mmol/l were found to be 1.74 times more likely to die than diabetic patients in the same range. Furthermore, non-diabetics were more than three times more likely to die compared with patients affected by diabetes when the time-weighted glucose concentration was between 10 and 11.1 mmol/l (11). A few years later the same authors observed a significant interaction between pre-existing hyperglycemia and the association of acute glycemia with mortality in ICU patients. High acute glucose concentration (>10 mmol/l) was significantly related to decreased hospital mortality only in patients with pre-admission HbA1c > 7% (12).

Regarding AIS patients, a historical systematic review by Capes et al. showed that stress hyperglycemia predicts increased risk of in-hospital mortality and poor functional recovery only in non-diabetics (24). In a further study, high levels of stress hyperglycemia, measured by the GAR index, increased more than twice the risk of stroke recurrence and all-cause death in AIS patients without diabetes (25). To date, only Chen et al. explored the influence of premorbid diabetic status on the relationship between stress hyperglycemia and functional outcome in AIS treated with MT. High levels of stress hyperglycemia were associated with an increased odd of poor outcome (OR 4.08, 95% CI 1.57–10.63, *p* = 0.004) in non-diabetics. On the contrary, diabetic patients with stress hyperglycemia did not show a similar risk (OR 2.75, 95% CI 0.68–11.11, *p* = 0.156) (16). Results of our study, conducted only in patients revascularized with IVT, are in the same line. In addition, in our sample the “modifier” role of premorbid diabetic status was observed not only for the functional outcome, but also for the other endpoint measures, i.e. mortality and hemorrhagic transformation.

Stress hyperglycemia impairs outcome in AIS patients by several mechanisms: (1) direct toxic damage due to lactate and intracellular acidosis (26); (2) stress-induced inflammatory response causing increased circulating free fatty acids (27, 28); and (3) reperfusion injury due to increased oxidative stress and inflammation (29). However, acute hyperglycemia seems to have different biological and clinical implications in diabetic patients treated with IVT, who showed a better outcome than non-diabetics. This could be explained by the fact that pre-stroke glucose-lowering therapy, taken by more than a half of our diabetics, reduces the amount of glucose able to diffuse into the brain, causing a decrease in cerebral lactic acidosis. Furthermore, insulin might limit the extent of the infarct through anticoagulant effects, such as reduced thromboxane production (30) and decreased plasminogen activator inhibitor-1 activity (31). Finally, it has been suggested that physiological cellular readjustment to higher glucose levels may occur in diabetic patients. Thus, higher glucose concentration might be necessary to exert its adverse effects among diabetic subjects (13).

A systematic literature review was recently performed to provide novel insights on glycemia control in AIS patients (32). The authors observed that, despite extensive active research, there is no standard approach to glycemia management. Regarding *when* to start hyperglycemia treatment, the 24 h window might be the optimal choice for treatment initiation. While, regarding hyperglycemia threshold requiring treatment, the authors agreed that it should be fixed at 180 mg/dl. Since post-stroke hyperglycemia shows a biphasic temporal pattern with an early phase (peaking at 8 h) and a delayed phase (peaking 48–88 h post-stroke), it is suggested to prolong treatment protocols for a minimum of 72 h (32). Recent studies, using continuous glucose monitoring devices, observed that glycemic variability, i.e. degree of fluctuation in glucose values over time, is associated with acute infarct growth and poor functional outcome in patients with acute stroke (33, 34). In particular, Palaiodimou et al. reported that greater glycemic variability of acute stroke patients was related to lower odds of neurological improvement during hospitalization (35).

Unfortunately, there is no clear consensus about the optimal therapeutic range of glucose values, that can be associated with better outcome of AIS patients. American guidelines for early management of AIS patients suggest to manage acute hyperglycemia to achieve blood glucose levels in a range of 140 to 180 mg/dl and prevent hypoglycemia (18). To date, several trials compared aggressive glycemic control vs. standard of care in acute stroke (36–39). The most recent of them, the SHINE trial, has been published in 2019. It included adult patients with hyperglycemia and recent AIS. Patients were randomized to receive aggressive glycemic control using continuous intravenous infusion of insulin (target blood glucose concentration of 80–130 mg/dl) or standard of care using sliding scale insulin treatment administered subcutaneously (target blood glucose concentration of 80–179 mg/dl). Patients were enrolled within 12 h of symptom onset and were maintained at either goal for the first 72 h of hospital admission. During treatment, the mean blood glucose level was 118 mg/dl in the intensive treatment group and 179 mg/dl in the standard treatment group. A favorable outcome, defined as a 3-month mRS score ranging between 0 and 2 on the basis of the NIHSS score at admission, occurred in 119 of 581 patients (20.5%) in the intensive treatment group and in 123 of 570 patients (21.6%) in the standard treatment group. Thus, this trial failed to demonstrate that acute hyperglycemia treatment with aggressive glycemic control is beneficial in AIS patients (39). Although episodes of severe hypoglycemia, occurring only among patients in the intensive treatment group, could explain these results (40, 41), a possible role of the premorbid diabetic status in modulating how acute glycemia interacts with clinical outcome in the SHINE trial cannot be excluded. In fact, 923 of the 1,151 patients (80%) enrolled in the trial were diabetics and, as previously reported (16, 24, 25), diabetic patients with AIS seem to be less affected by the detrimental effects of stress hyperglycemia than non-diabetic ones. Previous trials in ICU patients are in line with this hypothesis. The first Leuven trial, that randomized 1,548 surgical ICU patients to intensive glycemic control (goal glucose 80–110 mg/dl) vs. conventional glycemic control (180–200 mg/dl), observed that non-diabetic subjects sustained most of the survival benefit of intensive insulin therapy. In fact, mortality rates for the interventional and control arms were 4.7 vs. 8.4% among the non-diabetics and 4 vs. 5.8% among the diabetic subjects (6). A further study of 5,365 non-cardiac surgery patients admitted to ICU confirmed that only non-diabetics benefited greatly from tight glycemic control (10).

The results of this study are limited by several factors: (1) the retrospective design of the study may produce systematic error and bias; (2) the observed associations are no proofs of causality; thus, our results should be considered as hypothesis generating; (3) the tertile-based analysis might have affected the adequate control for confounding variables; (4) the relatively small sample size may have limited the statistical power; in particular, regarding the association of stress hyperglycemia with in-hospital mortality and hemorrhagic complications among diabetic patients.

In conclusion, premorbid diabetic status seems to influence outcome in AIS patients receiving IVT. In particular, odds of functional dependency, mortality and hemorrhagic complications were significantly increased in patients with more severe stress hyperglycemia only when they were not affected by diabetes. Our results, if confirmed by further larger prospective study, might be very useful in clinical practice. As hypothesized by Krinsley for critically ill patients (13), intensive insulin treatment might need to be tailored on the basis of the premorbid diabetic status in AIS patients treated with IVT and a tight glycemic control might be reserved only for non-diabetic patients.

## Data Availability Statement

The raw data supporting the conclusions of this article will be made available by the authors, without undue reservation.

## Ethics Statement

The studies involving human participants were reviewed and approved by Comitato Etico, Azienda Ospedaliero Universitaria, Udine. The patients/participants provided their written informed consent to participate in this study.

## Author Contributions

GM and SP: conceptualization. GM, SP, and YT: methodology. SP, YT, and SL: software. SL, GG, and MV: validation. GM: formal analysis, data curation, writing-original draft preparation, and writing-review and editing. SP, YT, SL, and AS: investigation and resources. GG: visualization. MV: supervision. All authors contributed to the article and approved the submitted version.

## Conflict of Interest

The authors declare that the research was conducted in the absence of any commercial or financial relationships that could be construed as a potential conflict of interest.

## Publisher's Note

All claims expressed in this article are solely those of the authors and do not necessarily represent those of their affiliated organizations, or those of the publisher, the editors and the reviewers. Any product that may be evaluated in this article, or claim that may be made by its manufacturer, is not guaranteed or endorsed by the publisher.
