# Supplementary material for: Stress Hyperglycemia Does Not Affect Clinical Outcome of Diabetic Patients Receiving Intravenous Thrombolysis for Acute Ischemic Stroke
Source: Front Neurol. 2022 Jun 13;13:903987. doi: 10.3389/fneur.2022.903987 (PMC9234697; doi:10.3389/fneur.2022.903987)
Supplement: Supplementary file 1 [file Data_Sheet_1.pdf]

Supplementary Figure 1. Rates of no major neurological improvement at discharge according to the GAR tertiles in diabetic (A) and non-diabetic patients (B)

A

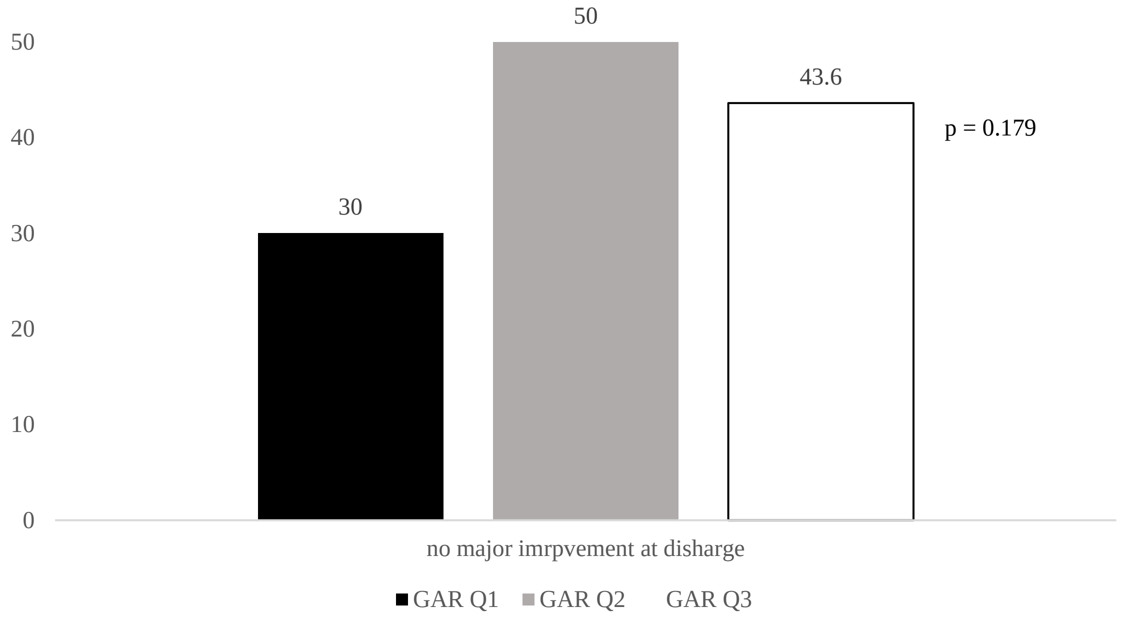

B

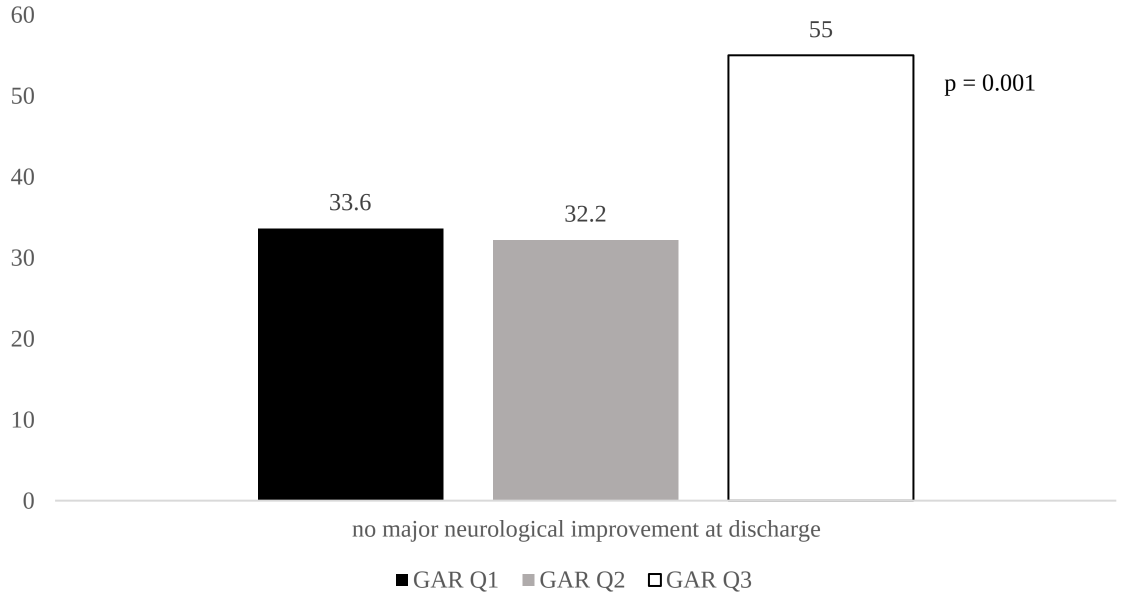

GAR Q1: first glucose-to-glycated hemoglobin ratio tertile; GAR Q2: second glucose-to-glycated hemoglobin ratio tertile; GAR Q3: third glucose-to-glycated hemoglobin ratio tertile.

Supplementary Figure 2. Rates of in-hospital mortality according to the GAR tertiles in diabetic (A) and non-diabetic patients (B)

A

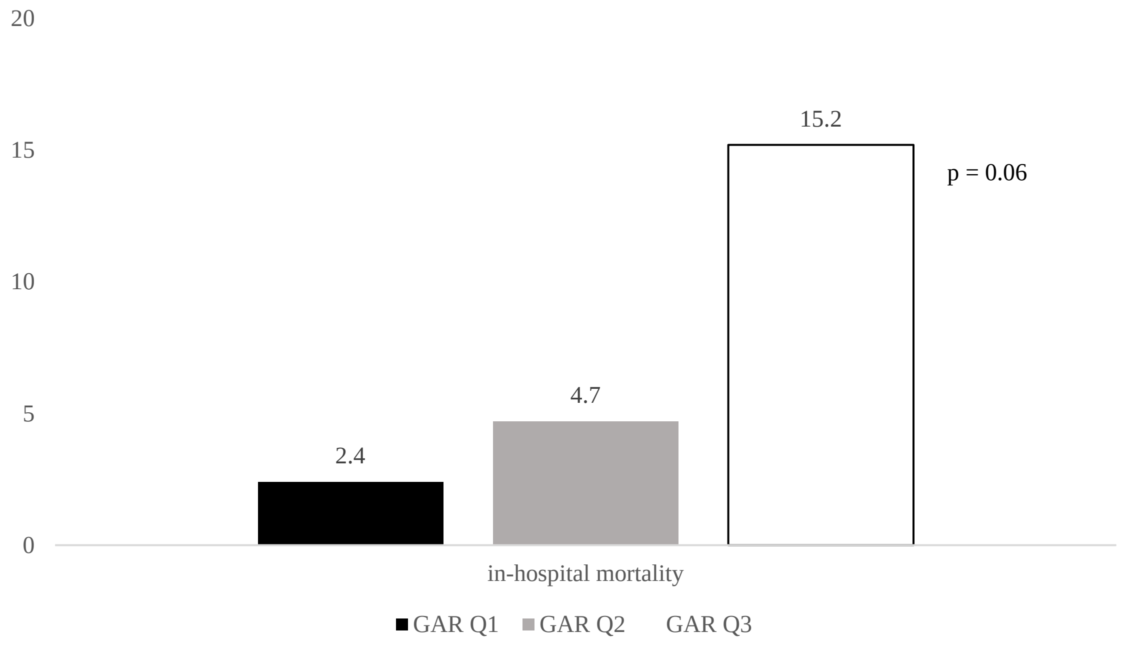

B

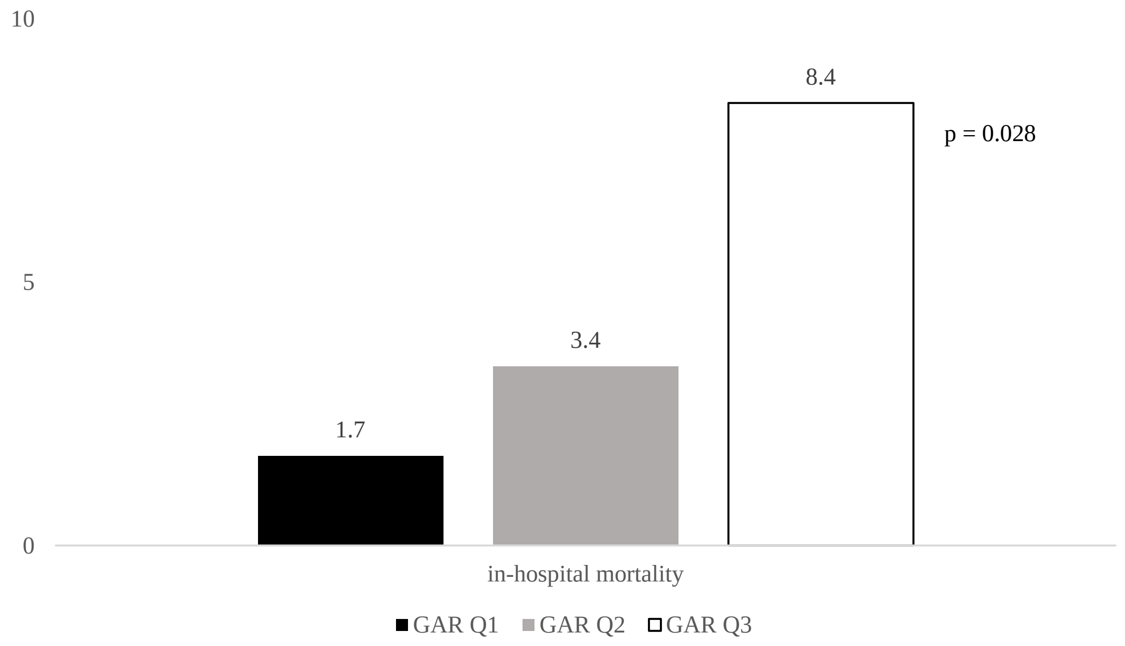

GAR Q1: first glucose-to-glycated hemoglobin ratio tertile; GAR Q2: second glucose-to-glycated hemoglobin ratio tertile; GAR Q3: third glucose-to-glycated hemoglobin ratio tertile.

Supplementary Figure 3. Rates of ICH according to the GAR tertiles in diabetic (A) and non-diabetic patients (B)

A

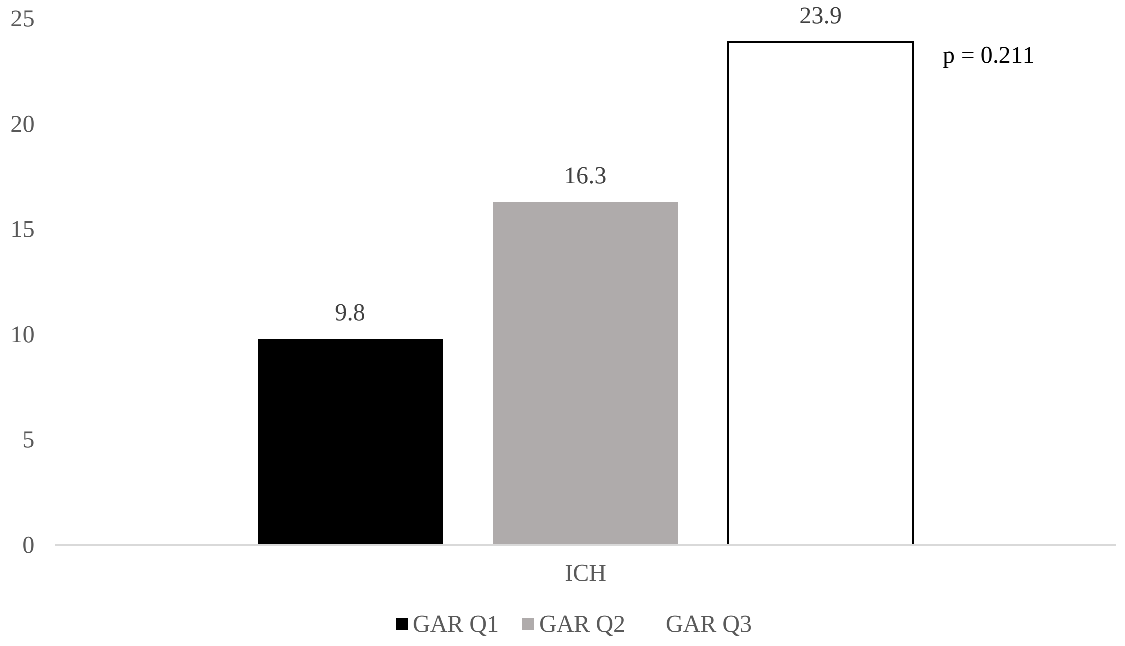

B

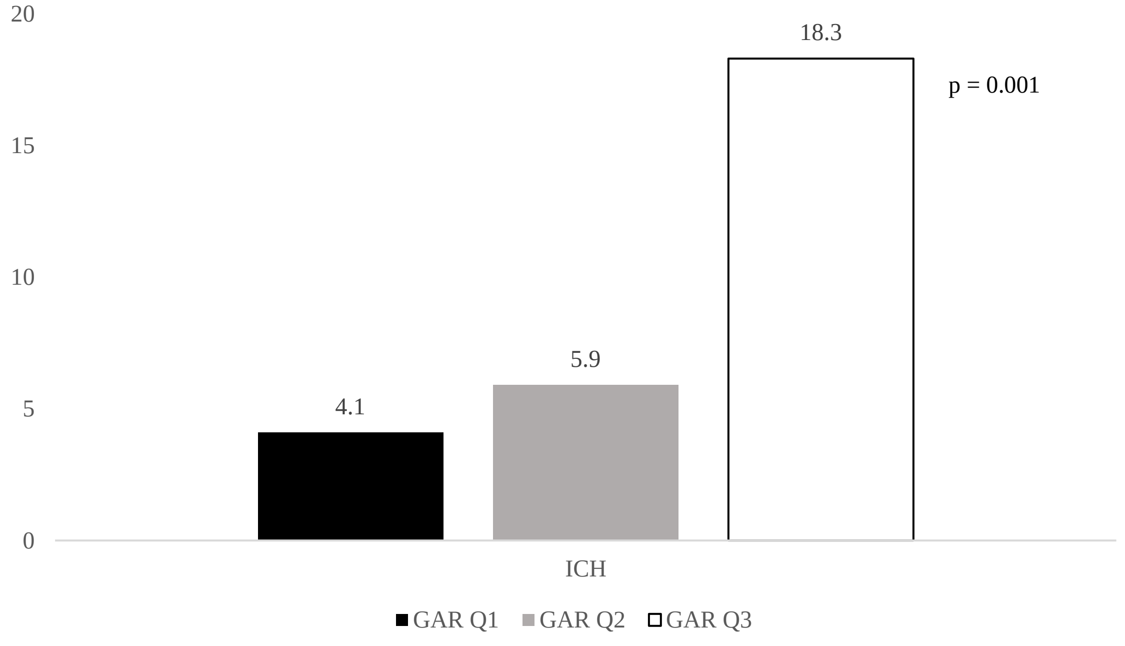

GAR Q1: first glucose-to-glycated hemoglobin ratio tertile; GAR Q2: second glucose-to-glycated hemoglobin ratio tertile; GAR Q3: third glucose-to-glycated hemoglobin ratio tertile; ICH: intracranial hemorrhage.
