# Supplementary material for: Stress Hyperglycemia Does Not Affect Clinical Outcome of Diabetic Patients Receiving Intravenous Thrombolysis for Acute Ischemic Stroke
Source: Front Neurol. 2022 Jun 13;13:903987. doi: 10.3389/fneur.2022.903987 (PMC9234697; doi:10.3389/fneur.2022.903987)
Supplement: Supplementary file 2 [file Data_Sheet_2.pdf]

Supplementary Table 1. Logistic regression model: adjusted ORs (95% CIs) of the GAR tertiles in relation to the respective secondary outcome measures in 130 diabetic patients and in 371 non-diabetic ones

	GAR Q1	GAR Q2	GAR Q3
**Diabetics, n = 130**			
No major neurological improvement at discharge†	1	2.4 (0.91-6.30) p = 0.076	1.63 (0.62-4.3) p = 0.322
In-hospital mortality††	1	1.27 (0.3-53.03) p = 0.9	18.29 (0.53-63.38) p = 0.107
Presence of ICH†††	1	2.14 (0.49-9.26) p = 0.309	2.99 (0.74-12.08) p = 0.123
**Non-diabetics, n = 371**			
No major neurological improvement at discharge*	1	0.94 (0.51-1.73) p = 0.834	2.11 (1.15-3.87) p = 0.016
In-hospital mortality**	1	2.88 (0.48-17.14) p = 0.244	5.19 (1.05-25.56) p = 0.043
Presence of ICH***	1	2.83 (0.47-17.05) p = 0.257	5.19 (1.05-25.61) p = 0.043

GAR Q1: first glucose-to-glycated hemoglobin ratio tertile; GAR Q2: second glucose-to-glycated hemoglobin ratio tertile; GAR Q3: third glucose-to-glycated hemoglobin ratio tertile; ICH: intracranial hemorrhage.

†Adjusted for: age, hypertension and stroke due to small vessel disease. ††Adjusted for: atrial fibrillation, hypercholesterolemia, C-reactive protein, triglycerides and baseline NIHSS score. †††Adjusted for: age, C-reactive protein, total cholesterol, stroke due to small vessel disease and baseline NIHSS score. *Adjusted for: age, C-reactive protein, HDL cholesterol, systolic blood pressure, baseline NIHSS score and pre-stroke mRS. **Adjusted for: age, sex, atrial fibrillation, baseline NIHSS score, pre-stroke mRS, time from symptoms onset to alteplase and door-to-needle time. ***Adjusted for: age, atrial fibrillation, triglycerides, ASPECTS, stroke due to cardioembolism, stroke due to small vessel disease, baseline NIHSS score and pre-stroke mRS.
